# Multiple Free Flap Reconstruction of a Complex Intraoral Defect after Squamous Cell Carcinoma Excision: A Case Report

**DOI:** 10.3390/medicina58010054

**Published:** 2021-12-30

**Authors:** Manlio Santilli, Gianmaria D’Addazio, Imena Rexhepi, Bruna Sinjari, Arnaldo Filippini

**Affiliations:** 1 Department of Innovative Technologies in Medicine and Dentistry, University “G. d’Annunzio” Chieti-Pescara, 66100 Chieti, Italy; santilliman@gmail.com (M.S.); gianmariad@gmail.com (G.D.); imena.rexhepi@gmail.com (I.R.); 2 Electron Microscopy Laboratory, University “G. d’Annunzio” Chieti-Pescara, 66100 Chieti, Italy; 3 Private Practice Surgeon, Malatesta Novello, 47521 Cesena, Italy; filippini@outlook.com

**Keywords:** free-flap, maxillary reconstruction, maxillectomy, oral cancer, fibular flap, radial flap

## Abstract

*Background:* Squamous cell carcinoma is the most frequent malignant cancer of the oral cavity. Metastasis involvement is one of the most relevant prognostic factors in terms of survival probability. Patients with oral cancers often undergo extensive *en bloc* resective surgery of the mandible and maxilla, with or without cervical nodal dissection, based on the presence or occult risk of regional metastases. Several factors affect the choice of flap, to recover aesthetics and function. *Case Presentation*: The case of a 60-year-old man who underwent maxillectomy with neck dissection as well as a reconstruction with a combination of multiple vascularized free flaps is presented. *Conclusions:* The excellent integration of the free flaps and the total absence of complications led to a high-quality aesthetic and functional performance of the reconstruction obtained through two different flaps. More specifically, the fibular free flap for bone reconstruction allows a two-team approach and maintains an excellent vascularization, even in case of several osteotomies for the maxillary reconstruction as reported. In addition, the use of free radial forearm flap for soft tissue reconstruction permits to obtain long caliber vessels, thus facilitating surgery without repositioning of the patient during surgery and therefore, consequently reducing surgery times.

## 1. Introduction

Squamous cell carcinoma is the most frequent malignant cancer of the oral cavity. Epidemiological data indicate that oral cancer is far from being rare [1]. In the last years, an increase of over 300,000 new oral squamous cell carcinoma (OSCC) cases has been recorded at global level. Moreover, a decrease in the average age and a higher number of female patients were reported [2]. Despite all efforts to prevent and treat the disease at an early stage, 50–60% of OSCC patients still die within five years [2,3]. A recent systematic review shows that—following successful treatments (five to 25 years)—the rate of metastatic spread for OSCC is about 65% for lymph node metastasis, 5–25% for distant one and up to 50% rate for local recurrence [4]. It is clear that metastasis involvement is one of the most relevant prognostic factors in terms of survival probability. Patients with oral cancers often undergo extensive *en bloc* resective surgery of the mandible and maxilla, with or without cervical nodal dissection, based on the presence or occult risk of regional metastases [3].

Therefore, having large safety margins is crucial to reduce local invasiveness and the possibility of the relapse of the disease. In advanced stages, the cancer may present ulcerations, a deep ulcer with an irregular vegetating surface, raised margins and a significant infiltrate of the oral tissues until the rapid involvement of lymph nodes and contributing to metastasis [5]. Maxillectomy defects often result in alterations in speech, chewing and swallowing due to the inability to separate the oral cavity from the nasal and paranasal cavities effectively. Therefore, this type of intervention results in important and extensive defects of soft tissues and/or bone with serious aesthetic and functional sequelae. For example, in partial and total maxillectomy it is very common to find cases of collapse of the lip, cheek and infraorbital soft tissue, loss of the hemipalate causing the above-mentioned alterations [6,7]. If the defect is not adequately reconstructed, a large oro-nasal and/or maxillary communication can be established. In order to recover aesthetics and function, numerous reconstructive options have been proposed over the years, among which revascularized bone flaps, pedunculated or revascularized flaps or free flaps. Many factors and variables influence the choice of flap type, such as the clinical condition of the patient, the size of the defect and its location. More specifically, the use of free revascularized flaps allows for a reliable repair in case of complex and extensive tissue defects, given that an abundant vascularization can reduce the infectious risks linked to the oral bacterial flora [8]. In addition, the bone tissue transplant which maintains its osteogenic properties can ensure a better osteointegration of the dental implants and permits a higher quality integration with the nativity mandibular bone [9]. The purpose of this case report is to present a partial maxillectomy with a double free flap vascularized reconstruction technique, in order to obtain the best reconstructive solution from a functional and aesthetic point of view.

## 2. Case Presentation

**Figure 1:**
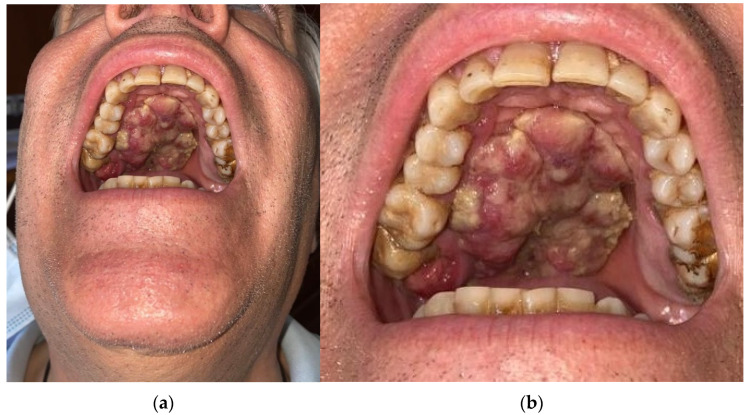
Clinical photo showing the intraoral aspect of the lesion. No extraoral swelling is present (**a**). Detail of clinical photo, revealing exophytic and ulcerated lesion with irregular aspect starting from the hard palate, 5 × 4 cm sized (**b**).

**Figure 2:**
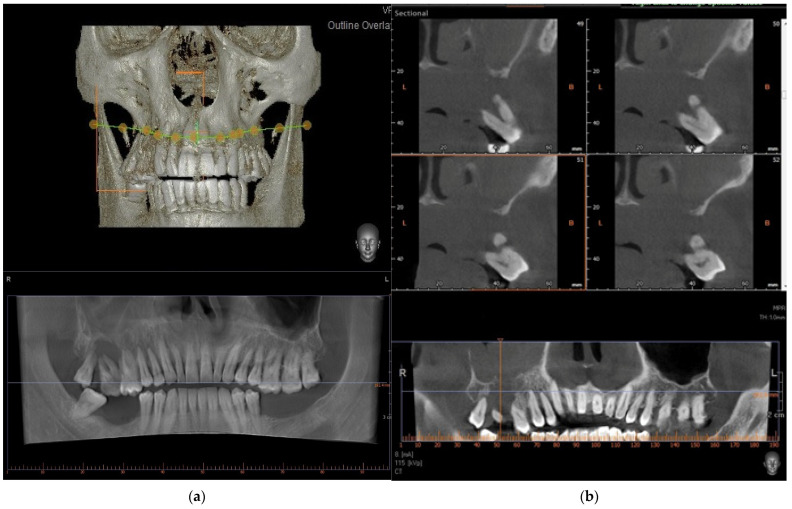
Preoperative radiograph images. In image (**a**) no bone destruction is detectable in the 3D reconstruction. Below, a simil panoramic image shows bone destruction in the upper right molar region (**a**). CBCT reveals the osteolytic change in the upper molar region, involving the hard palate (right) (**b**).

**Figure 3:**
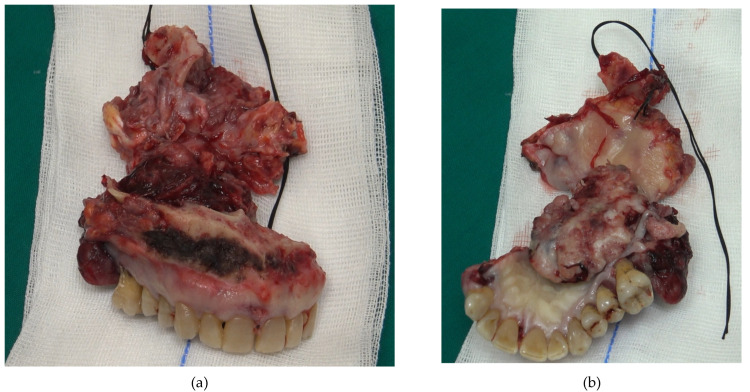
Anterior (**a**) and posterior (**b**) images of the surgical specimen after resection. The specimen is approximately 12 × 8 cm sized. Large margins of healthy tissue have been removed.

**Figure 4:**
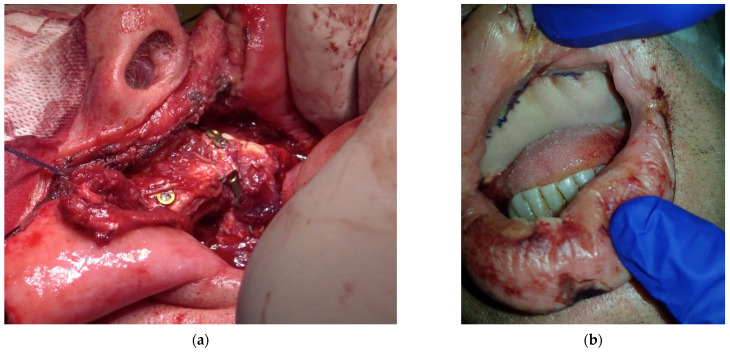
Intraoral images after hard tissue (bone) reconstruction with vascularized fibula free flap (**a**) and soft tissue reconstruction with vascularized free radial forearm flap (**b**).

**Figure 5:**
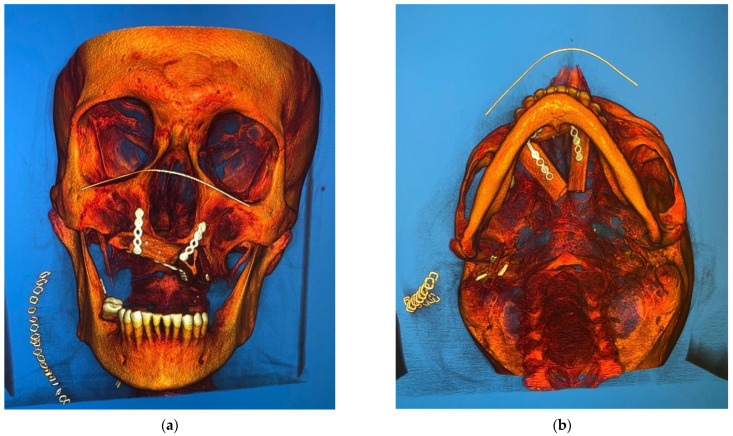
CBCT reconstruction after surgery (**a**-frontal view; **b**-coronal view). The CBCT was performed the day after surgery.

**Figure 6:**
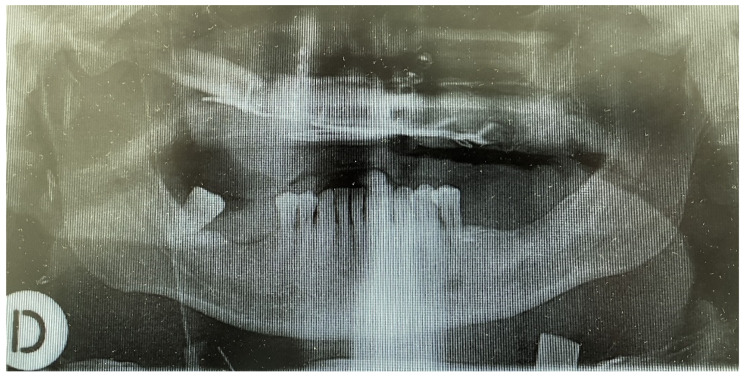
Post reconstruction panoral X-ray (The day after surgery).

A 60-year-old male patient, with dependence on alcohol and tobacco, was admitted to “Casa di Cura Malatesta Novello” (Cesena, Italy) for a not healing lesion on the hard palate. The patient gave a medical history of type 2 diabetes and multiple thrombophlebitis. He was under medication with metformin. The patient was examined both clinically and radiographically. Extraoral examination showed pain and difficulty in swallowing and phonation. Intraoral examination was difficult due to restricted and painful mouth opening, and it has shown an exophytic and ulcerated lesion, 5 × 4 cm sized and localized on the hard palate, having a warty and irregular aspect (Figure 1a,b). No Suspicious lymph nodes were noted extraorally. On palpation, bleeding was also evident. As shown in Figure 2, a cone beam computed tomography (CBCT) revealed extensive hard tissue destruction resulting from the hard palate. An incisional biopsy carried out in July 2020 revealed fragments with aspects of poorly differentiated squamous carcinoma. On the basis of the radiological, clinical and histological findings, a provisional diagnosis of oral squamous cell carcinoma was made. The patient was surgically treated on 24 September 2020. Surgery was performed under general anesthesia in the operating room. The multidisciplinary team included surgical oncologists, oral and maxillofacial surgeons, and otolaryngologist. Maxillectomy with neck block dissection of lymph nodes was performed (Figure 3). A reconstruction with fibula free flap for bone reconstruction (about 20 × 8 cm) and free radial forearm flap for soft tissue reconstruction (about 5 × 7 cm) was performed (Figure 4, Figure 5 and Figure 6). Complete resection of the lesion with clear margins was performed. The recipient vessels that were exploited for the fibula flap are the facial artery and the facial vein. For the free radial forearm flap, the superior thyroid artery and internal jugular vein were exploited.

A short video highlights all the surgical steps (please refer to Supplementary Materials section). Table 1 shows a description of the main surgical steps.

After excisional biopsy, histological diagnosis confirms poorly differentiated squamous cell carcinoma with massive latero-cervical metastases T_4_N_2_M_0_, based on mouth cancer TNM classification criteria of the American Joint Committee for Cancer Staging [10]. After histological diagnosis, the patient was subjected to cycles of chemotherapy and radiotherapy, as required by international protocols. The duration of postoperative follow-up was 13 months up to date. As demonstrated by clinical photos and post-operative radiographs, both free flaps were integrated in an excellent way. No postoperative complications emerged.

## 3. Discussion

There is wide evidence in the literature that, among others, the presence of lymph node metastasis and the invasion of resection margins represent the most important prognostic indicators of survival in the case of OSCC [11]. With the aim of obtaining optimal conditions to reach aesthetic and functional goals, the use of multiple microvascular free flaps is necessary [12,13]. Specifically, in the present case, multiple vascularized free flaps (fibula free flap for bone reconstruction and free radial forearm flap for soft tissue reconstruction) were used. The choice of the flap is guided by several criteria among which age, general health status, extension and site of the defect, status of dentition and others (as shown in Table 2) [12].

As for the bone, it is essential to analyze it both quantitatively and qualitatively, by taking into consideration previous surgical trauma also based on the patient’s age. As for the flap, it is necessary to assess the ease of collection with consequent site morbidity, definitely also the length of the vascular peduncle as well as the caliber of its vessels. It was demonstrated that both vascularized and non-vascularized techniques are well-accepted treatment strategies for reconstruction of the jaws, but double-flap procedures are frequently avoided because they are often associated with increased risk and higher complication rates [13,14,15,16]. The use of non-vascularized bone graft is not recommended in malignant disease and in defects >6 cm [17,18]. However, the approach with multiple free flaps has been chosen according to the evidence in literature showing that double-flap reconstructions can be realized with similar complications than the ones of single flaps [13]. Patients with free-flap reconstruction after oral cancer resection showed a five-year better trend survival, also in terms of quality of life [19]. Therefore, there are three main free flaps which are useful in case of hard tissue reconstruction for correct implant rehabilitation: iliac crest, scapula, and fibula. However, some cases of mandibular reconstructions with radium flap have been reported in the literature and, recently, also 3D reconstruction techniques are under development [20]. The latter allows to reduce the timing of surgery, the costs for operations as well as the time for general anesthesia, which is a key factor in the postoperative treatment. Moreover, accuracy, precision, and the ability to faithfully reproduce the bone to be replaced are the main features characterizing these techniques [20,21,22]. However, these techniques require excellent cooperation between surgeons, radiologists and a team of engineers, consequently increasing the time of the pre-surgical procedure and increasing the final cost of treatment. Therefore, free vascularized flaps remain the best option in case of extensive resection [22]. The iliac crest flap represents the gold standard for bone regeneration in young patients, specifically in terms of height for correct implant rehabilitation. This technique was used with trophic mandible and defect which includes the angle [12]. However, there is not a good predisposition of this flap for reconstruction of intraoral soft tissue defects. In fact, both the entire oblique muscle and the skin paddle are difficult to adapt to this type of reconstruction [9,12]. Regarding the scapula flap, it remains largely underutilized due to the location of the donor site. Generally, this is the most versatile technique for extensive defects, but on the other hand, it makes it impossible for two teams to work simultaneously, which prolongs operative time. In addition, the major drawback is represented by bone quality, due to the thickness of the tissue flap. Therefore, this is not suitable in case of extensive reconstruction [23,24]. The fibula free flap can be osseus or osteomyocutaneous. However, in this specific case the patient presented multiple thrombophlebitis; therefore, this type of flap was chosen only for bone reconstruction. The main advantage of this technique precisely lies in the anatomy of the bone itself, as a triangular-shaped bone which allows greater primary stability of the implants themselves, being a bicortical bone [25,26]. Moreover, one of the greatest strengths of the fibula free flap is that it can be manipulated without difficulty to fill defects on the inner and outer sides [27].

The fibula flap allows for a good reconstruction of intraoral soft tissue defects thanks to the skin flap’s great flexibility and mobility. On the other hand, in case of extensive reconstruction, the skin resulting from this type of flap is too small, thus making it necessary to consider other alternative solutions [23,24,25,26]. However, the criteria for choosing the type of flap to be used depend on the entity of the defect. In cases of bone defects <14 cm, the Iliac crest flap is preferred, while in defects >14 cm, the fibula flap is recommended [23,24,25,26]. Different flaps methods have been described for soft tissue reconstruction among which rectus abdominis free flap, pectoralis major free flap, free radial forearm flap, anterolateral thigh flap and others [28]. However, it is preferable to use the free radial forearm flap due to its flexibility [24]. For this reason, in the present case, the clinicians decided to use a fibula free flap for bone reconstruction, thus allowing to maintain an excellent vascularization, even if subjected to various osteotomies, and through a two-team approach. In addition, the clinicians decided to use also the free radial forearm flap for soft tissue reconstruction, used for intraoral defects (such as the palate defects) since this is mobile, thin and long (up to 20 cm), has a caliber vessel, thus facilitating surgery without repositioning of the patient during surgery, which decreases the surgical time [24]. Furthermore, the presence of thrombophlebitis also influenced the choice of this flap as described above. For this reason, in this specific case, the use of the anterolateral thigh flap was avoided [28]. After a 13-month follow-up, the excellent integration of the free flaps and the total absence of complications led to a high-quality aesthetic and functional performance of the reconstruction obtained via the use of two different flaps. Subsequently, it is planned to rehabilitate the patient with an implant prosthetic rehabilitation in order to obtain a total functional recovery of the chewing function.

It should be considered that the present study has some limitations. In fact, the follow-up time is not considerable since the surgery was performed approximately 13 months ago. In addition, it would be interesting to carry out a larger-sampled study to further understand whether the quality of life in patients with double vascularized free flaps reconstruction can improve.

## 4. Conclusions

The excellent integration of the free flaps and the total absence of complications led to a high-quality aesthetic and functional performance of the reconstruction obtained through two different flaps. More specifically, the fibular free flap for bone reconstruction allows a two-team approach and maintains an excellent vascularization, even in case of several osteotomies for the maxillary reconstruction, as reported. In addition, the use of free radial forearm flap for soft tissue reconstruction, allows to obtain long caliber vessels, thus facilitating surgery without repositioning of the patient during surgery and, therefore, consequently reducing surgery times.

## Figures and Tables

**Table 1:** Main surgical steps.

	Surgical Steps	
Timing (Hours)	Equipe 1	Equipe 2
0–1	General anesthesia and tracheotomy	
1–3	Maxillectomy	Fibula free flap
3–4	Neck block dissection of lymph nodes	Free radial forearm flap
4–7	Bone graft adaptation and osteosynthesis screw fixation	Donor site suturing
7–8	Soft tissue reconstruction	
8–10	Vascular micro-anastomosis	
10–11	General sutures	

**Table 2:** Main selection criteria for bone reconstruction. On the right side, three flaps are considered (Iliac, scapula and fibula); +++: First choice, ++: Highly Recommended, +: Recommended.

Selection Criteria for Bone Reconstruction	Iliac	Scapula	Fibula
Age (Young)	+++	++	++
Extension (>14 cm)	++	+++	+++
Localization donor site	+++	+	+++
Bone quality	+++	++	+++

## Data Availability

Not applicable.

## Supplementary Materials

The following supporting information can be downloaded at: https://drive.google.com/drive/folders/1NUIoPCrOGEYw-UMKFdgX4vpmYm58A_qB?usp=sharing accessed on 1 December 2021.
